# The role of AdhE mutations in *Thermoanaerobacterium saccharolyticum*

**DOI:** 10.1128/jb.00015-25

**Published:** 2025-04-30

**Authors:** João Henrique T. M. Fabri, Angel Pech-Canul, Samantha J. Ziegler, Tucker Emme Burgin, Isaiah D. Richardson, Marybeth I. Maloney, Yannick J. Bomble, Lee R. Lynd, Daniel G. Olson

**Affiliations:** 1 Centro de Biologia Molecular e Engenharia Genética (CBMEG), Universidade Estadual de Campinas (UNICAMP) 196609 , Campinas, State of São Paulo, Brazil; 2 Thayer School of Engineering, Dartmouth College 145792, Hanover, New Hampshire, USA; 3 Center for Bioenergy Innovation, Oak Ridge National Laboratory 6146, Oak Ridge, Tennessee, USA; 4 Biosciences Center, National Renewable Energy Laboratory 53405, Golden, Colorado, USA; 5 Terragia Corporation, Hanover, New Hampshire, USA; University of Florida Department of Microbiology and Cell Science, Gainesville, Florida, USA

**Keywords:** alcohol dehydrogenase, acetaldehyde dehydrogenase, AdhE, spirosome, substrate channeling, redox balance

## Abstract

*Thermoanaerobacterium saccharolyticum* is a thermophilic anaerobic bacterium that natively ferments a variety of hemicellulose substrates to organic acids and alcohols. It has recently been engineered to produce ethanol at high yield and titer; however, it uses a unique metabolic pathway for ethanol production that is poorly characterized. One of the distinctive aspects of this pathway is the presence of acetyl-CoA as an intermediate metabolite. In this organism, acetyl-CoA is converted to ethanol by a bifunctional AdhE enzyme. This enzyme has been a frequent target for mutations, and in many cases, the function of these mutations was unknown. Using a combination of genetic modifications, enzyme assays, and computational analysis, we have developed a better understanding of how mutations in AdhE affect ethanol production in the engineered homoethanologen strain. We identify a set of approximately interchangeable AdhE mutations (G544D, T597K, T597I, and T605I), whose function is to disrupt the activity of the alcohol dehydrogenase (ADH) domain of AdhE. This reduces NADH-linked ADH activity, which dramatically increases ethanol tolerance and changes the overall stoichiometry of acetaldehyde to ethanol conversion. Furthermore, our improved understanding of the function of these AdhE mutations calls into question a proposed feature of AdhE enzymes known as substrate channeling—direct transfer of acetaldehyde between the two domains of the AdhE enzyme. This improved the understanding of the role of AdhE mutations in *T. saccharolyticum* and provides deeper insights into the function of the unique ethanol production pathway in this organism.

**IMPORTANCE:**

Many anaerobic bacteria maintain redox equilibrium by producing reduced organic compounds such as ethanol. The final two steps of ethanol production are mediated by a bifunctional enzyme, AdhE, and this enzyme is a frequent target of mutations in strains engineered for increased ethanol production. Paradoxically, these mutations increase ethanol production by eliminating the activity of one domain of the AdhE enzyme (the ADH domain). This provides additional support for a redox-imbalance theory of alcohol tolerance, which challenges the prevailing hypothesis that alcohol tolerance is associated with cell membrane effects.

## INTRODUCTION

Lignocellulosic biomass—the fibrous material in terrestrial plants—is an abundant and renewable resource that could be used for sustainable production of fuels and chemicals. It is composed of cellulose, hemicellulose, and lignin (1). Consolidated bioprocessing (CBP) using engineered thermophilic bacteria has the potential to lower the cost of lignocellulose processing compared to the conventional paradigm involving thermochemical pretreatment and added cellulase (2–4). The thermophilic bacterium *Clostridium thermocellum* is more effective than a commercial cellulase preparation (e.g., Ctec 2) at deconstructing cellulosic biomass and has received considerable attention for use in CBP (5–9). Because *C. thermocellum* does not ferment hemicellulose or its derivatives, coculture with a compatible hemicellulose-fermenting thermophile has been proposed in order to convert all of the carbohydrate components present in lignocellulose (10–14).

One such hemicellulose-fermenting microbe is *Thermoanaerobacterium saccharolyticum*, a thermophilic organism that has the ability to ferment a wide range of sugars derived from lignocellulosic biomass (glucose, cellobiose, arabinose, mannose, galactose, xylose, and xylan) (15–17). Although the native organism is not highly ethanol-tolerant (growth is prevented at ethanol concentrations above ~20 g/L [18]), it has been engineered for ethanol production at high yield (> 90% of the theoretical maximum) (19) and high titer (~70 g/L) (20). Remarkably, this is one of only three organisms that do not natively ferment ethanol but have been engineered for high titer (> 60 g/L) production of ethanol by fermentation (the other organisms are *Escherichia coli* [21] and *Corynebacterium glutamicum* [22]). In strains of *T. saccharolyticum* engineered for high-yield ethanol production, mutations to the AdhE gene are commonly observed (19, 20, 23). However, the effect of these mutations and their resulting changes to pathway stoichiometry are not well understood.

There are at least three examples of AdhE mutations in *T. saccharolyticum*. In the first example, *T. saccharolyticum* was engineered by disruption of acetate and lactate production (19). The resulting homoethanologenic strain (named ALK2) had three mutations in its *adhE* gene: V52A, K451N, and a duplication of positions 649–661. Since the ALK2 strain included both of the available antibiotic resistance genes, no further genetic modifications were possible. To allow further modification, the strain was reconstructed, starting from the WT strain, using a marker recycling system (resulting in another homoethanologenic strain M0355) (24). This strain was subsequently adapted for improved growth in the presence of ethanol and other pretreatment inhibitors (25). Of the six strains that were studied, five had mutations in the *adhE* gene (G544D, S602L, and E603G), with some mutations occurring in more than one strain. Descendants of these strains include strain M1442, which has been shown to produce ethanol at a titer of 70 g/L, the highest reported for this organism (20). Finally, we engineered *T. saccharolyticum* for increased ethanol production by disrupting the *rex* redox-sensing transcription repressor gene. Here, we observed mutations either in or directly upstream of the *adhE* gene for all (4 of 4) strains tested (23). These mutations included T597K, T597I, and T605I. Despite this extensive set of mutations, only less is known about their effects. Much of the prior work characterizing the enzymatic activity has been performed in cell lysates, which makes it difficult to attribute the effects of mutations to a specific gene. Furthermore, some early measurements of ALDH activity did not include the required Mg cofactor (19, 26) and therefore do not accurately represent the ALDH activity present *in vivo* (see Hon et al. for a more detailed discussion [27]).

Furthermore, the stoichiometry of the *T. saccharolyticum* pyruvate to ethanol pathway is still uncertain. For each half glucose equivalent that is converted to acetyl-CoA, two electron carrier cofactors are reduced: NAD^+^ (from the glyceraldehyde-3-phosphate dehydrogenase reaction in glycolysis) and Fd_ox_ (from the pyruvate ferredoxin oxidoreductase reaction). Acetyl-CoA is then converted to ethanol by a series of two reactions, each of which oxidizes an electron carrier cofactor. The first reaction involves acetaldehyde dehydrogenase (ALDH), which oxidizes either NADH or NADPH. The second reaction involves alcohol dehydrogenase (ADH), which also oxidizes either NADH or NADPH (28). For high-yield ethanol production, the cofactors reduced in the first part of the pathway must match the cofactors oxidized in the final steps of the pathway. It is currently not known as to how electrons are transferred from reduced ferredoxin (Fd_red_) to NAD^+^ or NADP^+^. It is also not known which reduced cofactor (NADH or NADPH) is used for ALDH and ADH reactions. This has impeded attempts to transfer this pathway to other organisms, such as the cellulolytic bacterium, *Clostridium thermocellum* (27, 29–31).

In this work, we use a combination of genetic modifications and biochemistry to characterize the function of mutations in the *adhE* gene in *T. saccharolyticum* to better understand the stoichiometry of its pyruvate to ethanol conversion pathway.

## MATERIALS AND METHODS

### Media and growth conditions

All strains were grown anaerobically at 55°C. M122C rich medium at pH 6.5 was used for *T. saccharolyticum* transformation (32). For end-product analysis, *T. saccharolyticum* was grown in chemically defined MTC-6 medium (pH 6.5) (32). The MTC-6 medium was filter-sterilized immediately after preparation, transferred to sterilized serum bottles, and purged of oxygen using 20 45 second cycles of ultrahigh-purity N_2_ gas and vacuum. All fermentations were incubated for 120 hours in an orbital shaking incubator held at 55°C and 180 rpm. The fermentations were performed at least in triplicate in 125 mL glass serum bottles with a working volume of 50 mL and were inoculated with 1% frozen cell culture. To measure growth, cells were grown in 200 µL of MTC-6 medium in a 96-well plate, and the absorbance at 600 nm was monitored every 8 minutes for 96 hours.

### Construction of *T. saccharolyticum* strains

The strains used in this study are listed in Table 1. Primers used for the construction of the strains and confirmation of *adhE* manipulations are listed in Table S1. Point mutations were introduced into the chromosomal copy of the *adhE* gene by homologous recombination, using techniques that are described in detail in prior publications (32). In brief, *T. saccharolyticum* cells were transformed with cassettes containing the *adhE* gene followed by the kanamycin (kan) gene. These *adhE-kan* cassettes consist of four independent fragments: the non-mutated 5′ region of the *adhE* gene, a variable region of the *adhE* gene containing mutations (G544D, T597K, T597I, T605I, E603G, and a non-mutated WT control), the *kan* antibiotic resistance marker gene, and the downstream homology region of the *adhE* gene. The cassettes were constructed from linear PCR products via Gibson assembly (33), followed by anaerobic incubation with cells at 55°C to allow the naturally competent *T. saccharolyticum* cells to take up the DNA construct (34). The transformants were selected in plates with M122C rich growth medium supplemented with 200 µg/mL kanamycin.

**TABLE 1:** *T. saccharolyticum* strains used in this study

Strain	Description	Reference or source
LL1025	Wild-type *T. saccharolyticum* strain JW/SL-YS485	GenBank accession no. CP003184
ALK2	∆*ldh*::*erm* ∆(*pta-ack*)::*kan*^r^	(19)
LL1287	M1442 Δ*adhA*::*kan*^r^	(35)^*a*^
M1442	LL1025 Δ*pta* Δ*ack* Δ*ldh adhE*^G544D^. Also known as LL1049	(20) SRA accession no. SRA233073
A2G0001	M1442 *adhE*^G544D^::*adhE*^T597K^-*kan*^r^	This work
A2G0002	M1442 *adhE*^G544D^::*adhE*^T597I^-*kan*^r^	This work
A2G0003	M1442 *adhE*^G544D^::*adhE*^T605I^-*kan*^r^	This work
A2G0004	M1442 *adhE*^G544D^::*adhE*^E603G^-*kan*^r^	This work
A2G0022^*b*^	LL1025 *adhE*^WT^::*adhE*^WT^-*kan*^r^	This work
A2G0023	LL1025 *adhE*^WT^::*adhE*^G544D^-*kan*^r^	This work
A2G0024	M1442 *adhE*^G544D^::*adhE*^WT^-*kan*^r^	This work
A2G0025^*b*^	M1442 *adhE*^G544D^::*adhE*^G544D^-*kan*^r^	This work

^*a*^ The genome of this strain has not yet been re-sequenced, so no sequence accession number is available.

^*b*^ A2G0022 and A2G0025 are control strains to identify any polar effects of the kan marker without changing the *adhE* gene.

### Metabolite quantification

Ethanol and other fermentation products in the liquid phase (cellobiose, lactate, acetate, and formate) were measured using a Shimadzu LC-2050C high-pressure liquid chromatography (HPLC) instrument with an HPX-87H column and an additional UV detector (for the accurate quantification of pyruvate). The column was incubated at 60°C, and the mobile phase flow rate was 0.6 mL/min. Acetaldehyde was measured by derivatization with 2,4-dinitrophenylhydrazine and quantification by HPLC, as described previously (36, 37). All fermentation data are reported in Table S2.

### Ethanol tolerance assay

Ethanol tolerance was determined as described in (38). Briefly, assays were performed in a 96-well plate with a ThermalSeal RTS Sealing Film (Sigma), and the inoculum was prepared by inoculating 2 µL of fresh cells in 198 µL of MTC-6 media containing various concentrations of ethanol. Cell growth was determined by measuring the absorbance at 600 nm in 8 min intervals for 96 hours. The specific growth rate was determined by measuring the maximum slope of the log-transformed and blank-subtracted absorbance data. All ethanol tolerance data are reported in Table S3.

### AdhE cloning, protein expression, purification, and activity

AdhE cloning, protein expression, purification, and activity assay measurements for ALDH and ADH activity were performed exactly as described in our recent paper (37).

All chemicals were purchased from MilliporeSigma, unless otherwise noted. All enzyme assays used a buffer consisting of the following components: 100 mM Tris-HCl buffer, pH 7.5, 0.01 mg/mL bovine serum albumin (BSA, Thermo Scientific part number 23209), 250 mM NaCl (Fisher catalog number S271), 2 mM MgCl_2_ (Sigma catalog number M2670), 1 mM dithiothreitol (DTT, Sigma catalog number 43816), 10 mM sodium ascorbate (Sigma catalog number 11140), and 0.5 mM ammonium ferrous sulfate (Sigma catalog number 9719). All enzymes were assayed at 40°C.

ALDH activity was assayed in the physiological (acetaldehyde-forming) direction using the following assay. In addition to the standard buffer mixture (above), the reaction mixture contained the following: 0.45 mM NADH (Sigma catalog number N8129) or NADPH (Sigma catalog number N7505) and 1 mM acetyl-CoA (Sigma catalog number A2056). The reaction was started by adding acetyl-CoA. The reaction volume was 60 µL. Reactions were performed in a 384-well microplate. For the ALDH reaction, one unit (U) of activity corresponds to consumption of 1–2 umol of the substrate (NADH or NADPH) per minute for the ALDH reaction. The uncertainty of the stoichiometry of the ALDH reaction is due to the uncertainty of the efficiency of acetaldehyde transfer between the ALDH and ADH domains. For our calculations, we have assumed a stoichiometry of 1.

ADH activity was assayed in the physiological (ethanol-forming) direction using the following assay. In addition to the standard buffer mixture (above), the reaction mixture contained the following: 0.45 mM NADH or NADPH and 10 mM acetaldehyde (Sigma catalog number 402788). The reaction was started by adding acetaldehyde. The reaction volume was 60 µL. Reactions were performed in a 384-well microplate. For ADH activity, one unit (U) corresponds to consumption of 1 µmol of the substrate (NADH or NADPH) per minute.

### Structural analysis

The *T. saccharolyticum* AdhE monomer was modeled using AlphaFold 2 (39) with an input sequence of the wild-type AdhE. AlphaFold 2 computed high confidence in the modeled structure, which is unsurprising given the availability of crystal structures for closely related proteins. Four monomers were aligned to the *C. thermocellum* AdhE spirosome (PDB ID 8UHW) with an RMSD of <1 Å per subunit to form a theoretical spirosome. The NADH in each catalytic pocket was aligned to the structure via the *E. coli* spirosome structure (PDB ID 7BVP). Wild-type and mutant structures were analyzed in ChimeraX (40). Mutant rotamers were selected to minimize clashing. If all rotamers had clashes, the most prevalent rotamer was chosen for analysis.

### Molecular Dynamics simulations

Molecular dynamics (MD) simulations were performed using Amber 20 (41). The initial wild-type structure of a single AdhE monomer was obtained in the same manner as described above for structural analysis. Because our simulations contained only mutations in the ADH domain, we truncated the AdhE protein at residue 450, discarding the ALDH domain. The resulting model was protonated at pH 6.5 using the H ++ web server (42) and then prepared for simulation using the ff19SB protein force field (43), with previously published parameters for NADH (44, 45) and an octagonal box of OPC3 waters (46) with at least 8 Å between the protein and the edge of the box on each side. The desired mutations for each variant were applied using PyRosetta (47), protonation states were reassessed using PROPKA 3 (48), and then each system was energy-minimized over 5,000 steps and heated at constant volume from 100 to 333.15 K over 11,000 2-fs steps. Hydrogen mass repartitioning (49) with a factor of 3 was applied in order to facilitate longer MD time steps, and then three repeats each of 400-ns-long simulations at constant isotropic pressure with a 4-fs timestep were run for each variant. A non-bonded cutoff of 8 Å was used for all simulations, and during NVT and NPT simulations, the temperature was maintained using the Andersen thermostat (50) with an interval of 1,000 steps.

## RESULTS

### Enzymatic activity in cell lysates

**Fig 1:**
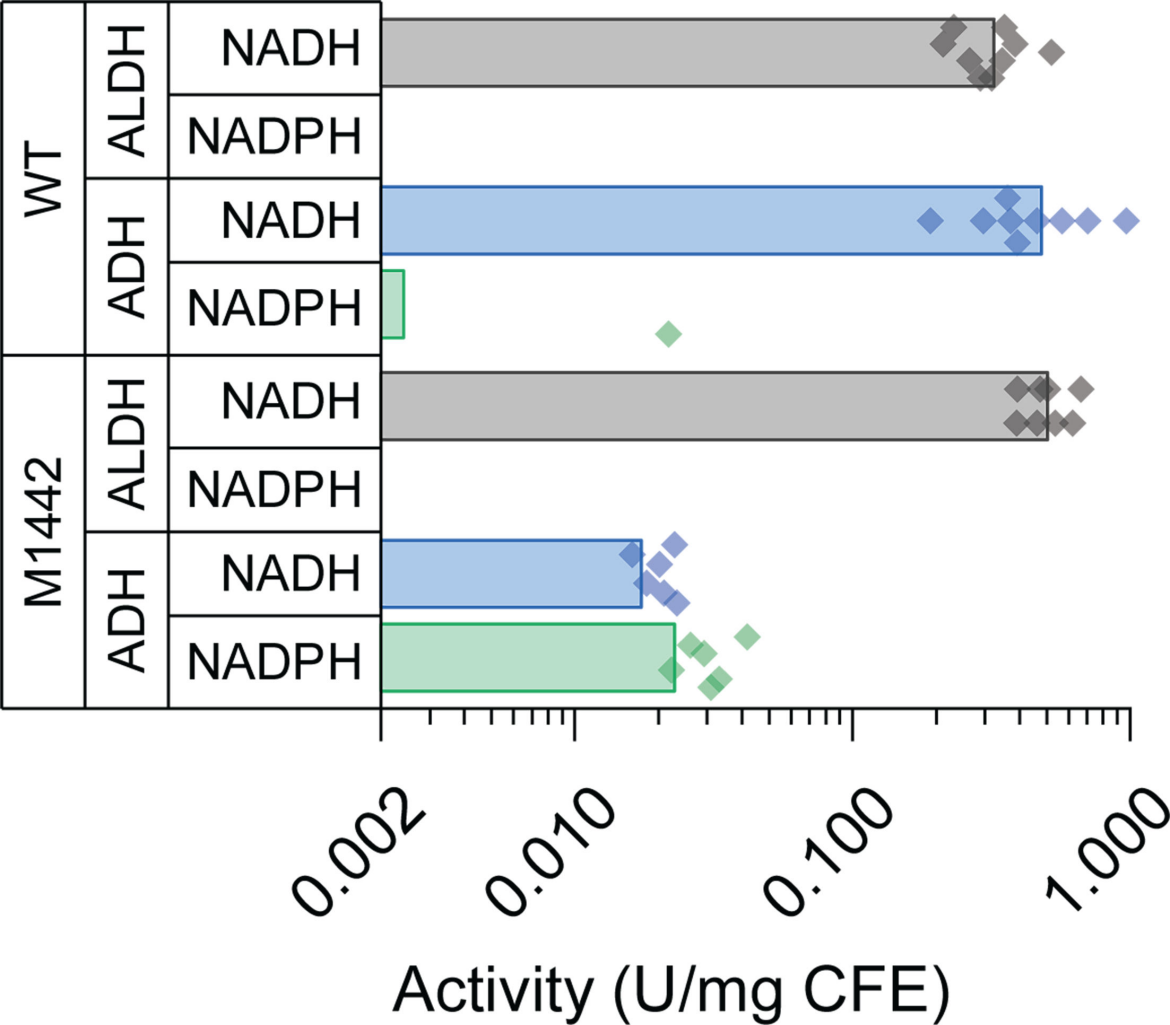
Enzymatic activity in cell-free extracts (CFE). Extracts were assayed for both ALDH activity (acetyl-CoA +NAD(P)H → acetaldehyde +CoA + NAD(P)^+^) and ADH activity (acetaldehyde +NAD(P)H → ethanol +NAD(P)^+^) using either NADH or NADPH as cofactors, respectively. Bars represent the average activity (U/mg CFE), where one unit (**U**) corresponds to the formation of 1 µmol of product per minute. Each data point represents the average activity from several dilutions of a single-cell lysate. Each strain was assayed at least six times. The absence of data indicates that the measured activity was below the limit of detection (~0.003 U/mg CFE for NADH-linked activity and ~0.01 U/mg CFE for NADPH-linked activity). No NADPH-linked ALDH activity was observed for either strain.

Strain M1442 (also known as strain LL1049) is one of the highest-titer ethanol-producing strains of *T. saccharolyticum* (20). Although we have previously measured the enzymatic activity in cell lysates, the ALDH assays did not include Mg, so we wanted to repeat them under more physiologically relevant conditions. Both the WT and M1442 strains exhibited only NADH-linked ALDH activity (Fig. 1). For ADH activity, the WT strain had mostly NADH-linked activity. In the M1442 strain, overall ADH activity decreased substantially; however, the remaining less ADH activity allowed use of either the NADH or NADPH cofactor (Fig. 1).

### Effect of mutations on the enzymatic activity

Since it is not possible to attribute the ADH activity to a single enzyme in cell lysates, we next measured the activity using purified enzymes cloned and expressed in *E. coli*. In addition to the G544D mutation we were most interested in (due to its presence in the high-ethanol-titer strain M1442), we also characterized 10 other mutations that had been previously observed in *T. saccharolyticum* AdhE (Table 2). For each enzyme, we measured both ALDH and ADH activities with both NADH and NADPH cofactors. Neither the WT nor any of the mutant AdhE enzymes showed substantial NADPH-linked activity for either the ALDH or ADH domains. Several of the mutant AdhE enzymes exhibited substantial decreases in the ADH activity (G544D, T597I, T597K, S602L, T605I, and the duplication of positions 649–661 (dup)). These mutations had variable effects on ALDH activity, with some mutations reducing the ALDH activity substantially (K451N, T597I, S602L, and T605I) and others having a more modest effect on ALDH activity reduction (G544D, T597K, E603G, and dup).

**TABLE 2:** *T. saccharolyticum* AdhE mutations

Mutation	Description	DNA seq. accession^*a*^	Reference or source
WT	WT *T. saccharolyticum* AdhE, no mutations	KR632761.1	(16)
V52A^*b*^	Originated in strain ALK2, engineered for increased ethanol production	KR632760.1	(19)
T183A	Mutation unintentionally introduced during cloning	PQ778317	This work
K451N^*b*^	Originated in strain ALK2, engineered for increased ethanol production	KR632760.1	(19)
G544D	Originated in strains M0734 and M0863, engineered for increased ethanol production	KR632762.1	(25)
T597I	Originated in *rex* deletion strain RexAdp-2 with reduced ethanol production.	SRP112528	(23)
T597K	Originated in *rex* deletion strain RexAdp-2 with reduced ethanol production.	SRP112528	(23)
S602L	Originated in strain M0699, engineered for increased ethanol production	PQ778318	(25)
E603G	Originated in strains M0694 and M0700, engineered for increased ethanol production	PQ778319	(25)
T605I	Originated in *rex* deletion strain RexAdp-5 with reduced ethanol production.	SRP112523	(23)
Duplication of positions 649–661^*b*^	Originated in strain ALK2, engineered for increased ethanol production	KR632760.1	(19)

^*a*^ Numbers starting with KR are GenBank accession numbers for *adhE* genes. Numbers starting with SRP are whole-genome sequences of *T. saccharolyticum* that include the *adhE* gene from the NCBI Sequence Read Archive (https://www.ncbi.nlm.nih.gov/sra).

^*b*^ The *adhE* gene in *T. saccharolyticum* strain ALK2 contains three mutations: V52A, K451N, and a duplication at positions 649–661.

**Fig 2:**
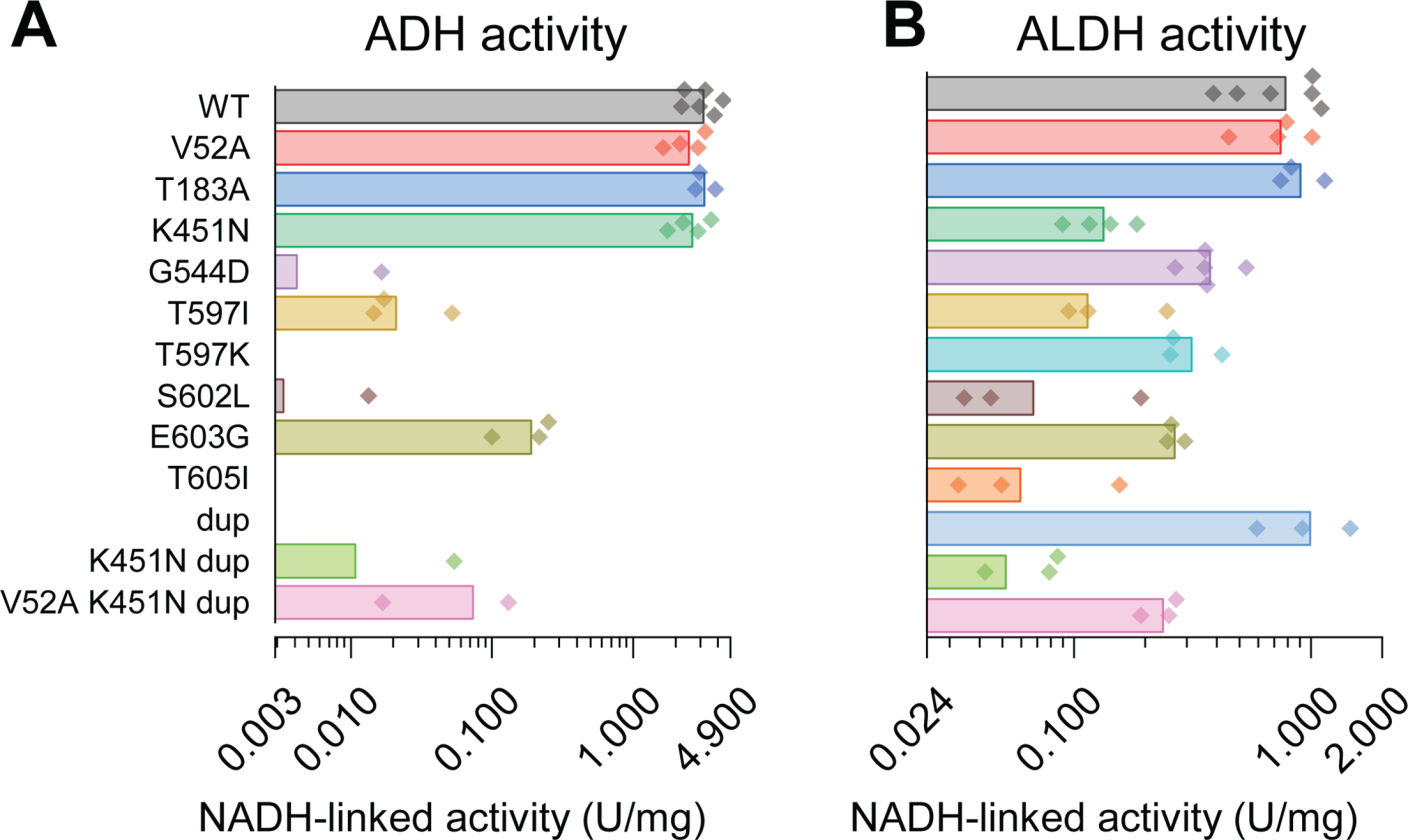
Enzymatic activity of mutant AdhE enzymes. Enzymes were cloned and expressed in *E. coli* and then purified by affinity chromatography. Each purified enzyme was assayed for both (**A**) ADH activity (acetaldehyde +NAD(P)H → ethanol +NAD(P)^+^) and (**B**) ALDH activity (acetyl-CoA +NAD(P)H → acetaldehyde +NAD(P)^+^), using either NADH or NADPH as cofactors (i.e., four separate assays for each protein). Bars represent the average activity (U/mg purified protein). Each data point represents the average activity from several dilutions of a purified AdhE protein. Each protein was assayed three to six times. The absence of data indicates that the measured activity was below the limit of detection (~0.003 U/mg protein for NADH-linked activity and ~0.01 U/mg protein for NADPH-linked activity). No NADPH-linked activity was observed for any of the mutants for either ALDH or ADH activity. All of the mutations are single-amino acid substitutions, except for the mutation labeled “dup,” which is a duplication of the amino acids at positions 649–661 (see Table 2 for more details).

We also looked at the effect of three mutations that appear together in another homoethanologenic strain of *T. saccharolyticum* ALK2 (19): V52A, K451N, and dup. Individually, the V52A mutation seems to have no effect, the dup mutation primarily reduces ADH activity, and the K451N mutation slightly reduces ALDH activity. Combining these mutations appears to restore a bit of the activity lost due to the dup mutation (Fig. 2).

### Effect of mutations on ethanol production and growth

To better understand the physiological significance of these mutations, we reintroduced several of these mutations into either the WT or homoethanologenic strain (M1442) background and measured the effect on growth and ethanol production. The WT strain can prepare a variety of fermentation products, so we hypothesized that reducing the AdhE activity might change the distribution of products or slow growth. Strain M1442 can only produce ethanol, so although we would not expect to see a change in the distribution of fermentation products, we might expect a change in the growth rate.

**Fig 3:**
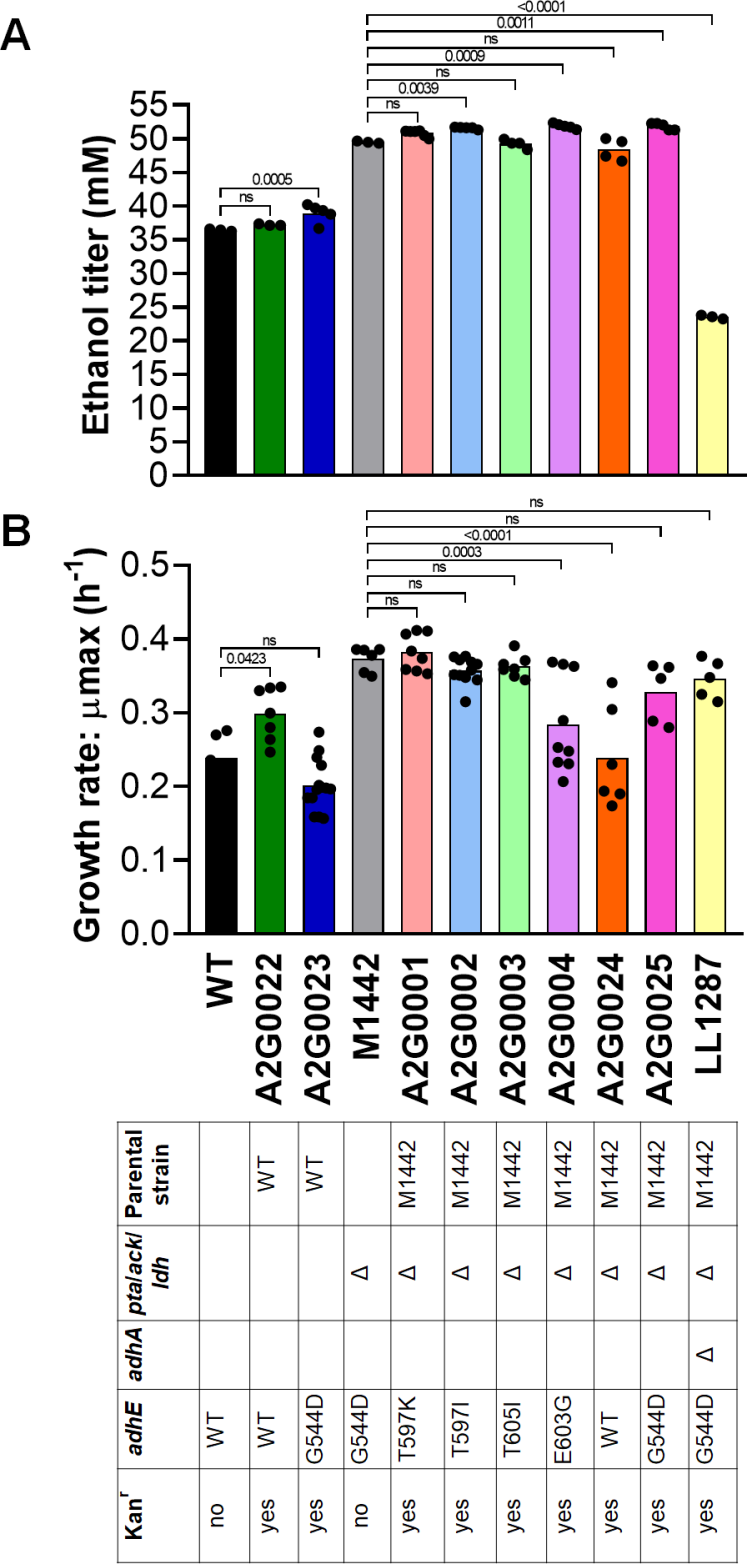
Ethanol titers and growth rates of *T. saccharolyticum adhE* mutants. All the strains were cultivated on the MTC-6 defined medium with 5 g/L cellobiose (14.7 mM). (**A**) Fermentations were carried out for 120 hours. (**B**) For the growth curves, the strains were cultivated in a 96-well plate containing the MTC-6 medium for 96 hours. Dots indicate the biological replicates. Statistical analysis was made using one-way ANOVA and Dunnett’s post-test in relation to wild-type (LL1025) or homoethanologenic (M1442) strains. The genotype of each strain is shown in the table below the graph, where blanks indicate the WT allele and Δ indicates disruption of the gene either by deletion or replacement with a *kan* marker. Introduced *adhE* mutations are also shown. Full genotypes of the strains are listed in Table 1. Complete fermentation data are available in Supporting Table S2.

In the WT strain background (strain LL1025), replacing the chromosomal *adhE* gene with either *adhE-kan* (control for the effect of the *kan* gene, strain A2G0022) or *adhE^G544D^-kan* cassettes (strain A2G0023) had no effect on ethanol production or growth rate (Fig. 3).

In the homoethanologen strain background (strain M1442), replacing the chromosomal *adhE^G544D^* gene with *adhE^mut^-kan* cassettes (where mut is one of six *adhE* mutations, i.e., WT, G544D, T597K, T597I, E603G, or T605I) had no effect on the ethanol titer (Fig. 3) or yield (Supporting Table S2). The effect on the growth rate was more variable. For the G544D, T597L, T597I, and T605I mutants, the growth rate was indistinguishable from the parent strain. For both the WT allele and the E603G mutant, some colonies showed slow growth, and some showed growth similar to that of the parent strain. For the WT allele, we suspect that the slow-growing colonies represent the true effect of the WT allele, and the faster-growing colonies have acquired new *adhE* mutations, based on the difficulty creating these strains and our frequent observation of mutations (Table S4).

Note that in this strain (M1442), the chromosomal *adhE* locus already contains the G544D mutation. Therefore, the reintroduction of the G544D mutant (as opposed to the WT allele) serves as a control for the effect of the downstream *kan* marker.

Our observation that ethanol production is uncorrelated with reduced ADH activity in purified AdhE proteins (Fig. 2) suggests that AdhE is not, in fact, playing a role in the conversion of acetaldehyde to ethanol in the homoethanologen strains (M1442 and its descendants). Instead, it appears that AdhA is the primary ADH enzyme in this strain since its deletion (Fig. 3—compare strain LL1287 (Strain M1442 with *adhA* deleted) vs the M1442 parent strain) has a much larger effect on ethanol production, consistent with our previous observations (35). To better understand the fate of substrate carbon (i.e., cellobiose) that was not converted to ethanol, we performed a batch fermentation with strains M1442 and LL1287 (Supporting Information Fig. S1). As expected, strain LL1287 grew more slowly than strain M1442. A substantial fraction of the cellobiose substrate was converted to glucose. We also observed increased acetaldehyde accumulation in the supernatant of strain LL1287 (11 mM vs 6 mM for the M1442 parent strain), providing additional evidence of the role of AdhA in conversion of acetaldehyde to ethanol.

### Effect of mutations on ethanol tolerance

**Fig 4:**
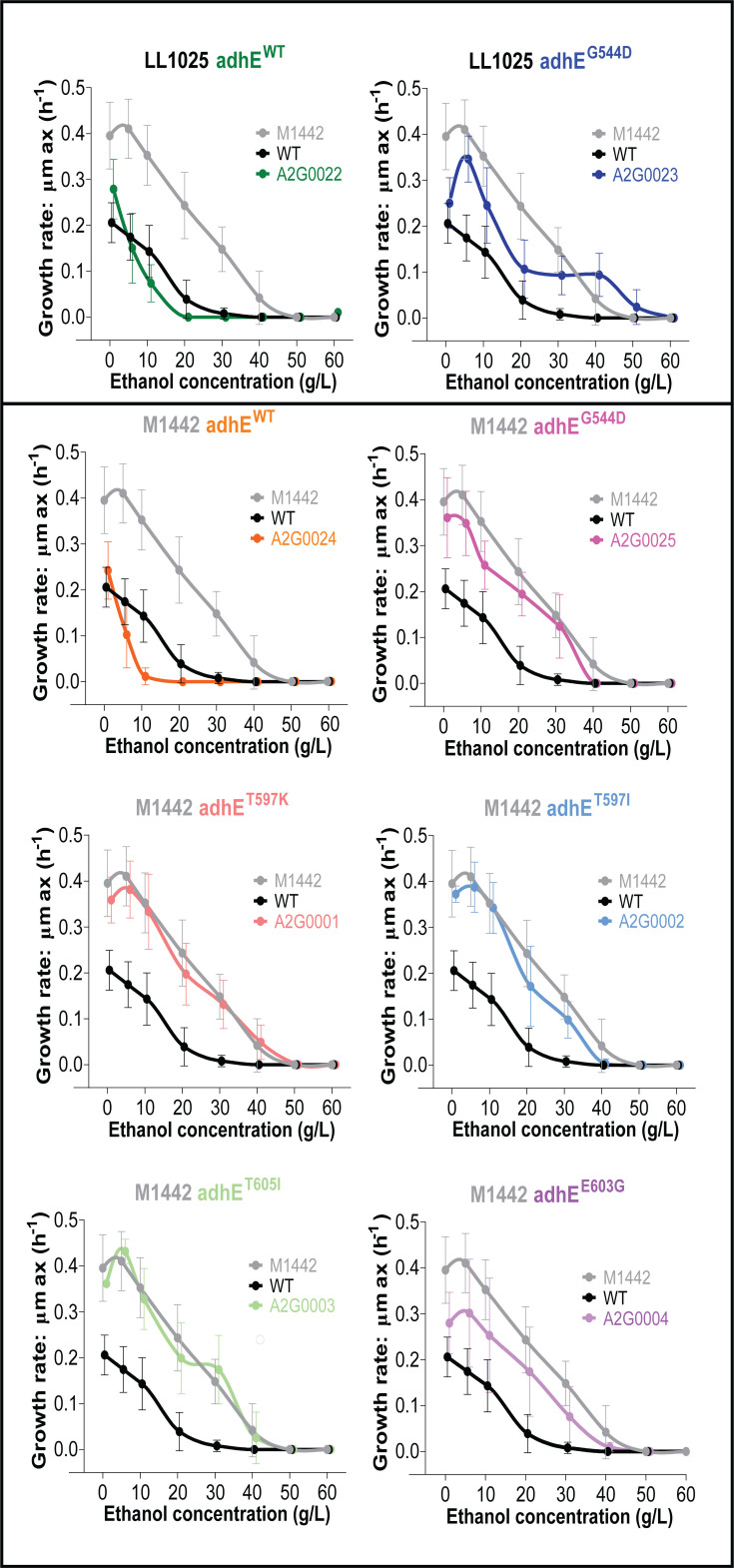
Ethanol tolerance of *T. saccharolyticum adhE* mutants. The growth rate was measured in a 96-well plate with various concentrations of added ethanol in the MTC-6 medium. In each panel, the WT (LL1025 strain—low ethanol tolerance) and homoethanologenic (M1442 strain—high ethanol tolerance) controls are included for reference (black and gray curves, respectively). Each panel is labeled according to the mutant being tested. For clarity, panel labels include the parent strain (LL1025 or M1442) and the introduced *adhE* mutation, color-coded to match the corresponding strain. Error bars represent one standard deviation with *n* ≥ 3 biological replicates. Data points were fitted with Akima spline/LOWESS on GraphPad Prism and are slightly offset along the x-axis to more clearly show error bars.

Next, we measured the effect of different AdhE mutations on ethanol tolerance. The role of AdhE on ethanol tolerance has been well-established for *Clostridium thermocellum* (37, 38, 51, 52) (another anaerobic thermophilic bacterium belonging to the class Clostridia—the same class as *T. saccharolyticum*), but not yet for *T. saccharolyticum*. During the development of one homoethanologenic strain of *T. saccharolyticum* (M1442), the strain was subjected to chemical mutagenesis, followed by selection for growth in the presence of various inhibitors, including ethanol (20). We, therefore, suspected that it would exhibit increased ethanol tolerance compared to the WT strain (LL1025), and this is what we observed (Fig. 4—compare WT with the M1442 strain).

To understand how mutations in the *adhE* gene affect ethanol tolerance, we introduced either the *adhE-kan* cassette (control for effects of the *kan* marker) or the *adhE^G544D^-kan* cassette into the WT strain (LL1025) at the *adhE* locus. Reintroduction of the WT allele had no effect on ethanol tolerance, and introduction of the G544D allele slightly increased ethanol tolerance (Fig. 4 upper panel).

We also performed a complementary experiment where we introduced mutant *adhE* genes into the homoethanologen strain (M1442). In this strain background, replacing the *adhE^G544D^* allele with the *adhE^WT^* allele reduced ethanol tolerance (Fig. 4). The G544D (control), T597K, T597I, or T605I mutant *adhE* genes had no effect on ethanol tolerance. The E603G mutant *adhE* gene exhibited slightly reduced ethanol tolerance compared to the M1442 parent strain (harboring the G544D mutation). Thus, in strain M1442, we observed a relatively consistent pattern of ethanol tolerance. The strains with the lowest levels of NADH-linked ADH activity (harboring the G544D, T597K, T597I, and T605I *adhE* mutations) had the highest ethanol tolerance. The strain with the highest levels of NADH-linked ADH activity (harboring the WT *adhE* allele) had the lowest ethanol tolerance. And a strain with an intermediate level of NADH-linked ADH activity (harboring the E603G mutation) had an intermediate level of ethanol tolerance. Of note, almost all M1442-derived strains appear to grow better in the presence of 5 g/L (11 mM) ethanol than in its complete absence. It is possible that as the M1442 strain went through selective pressure during the process of becoming ethanologenic, this caused better adapted growth at low ethanol concentrations, which was reflected in the other mutant strains with no change in ethanol tolerance.

### Structural effects of AdhE mutations

**Fig 5:**
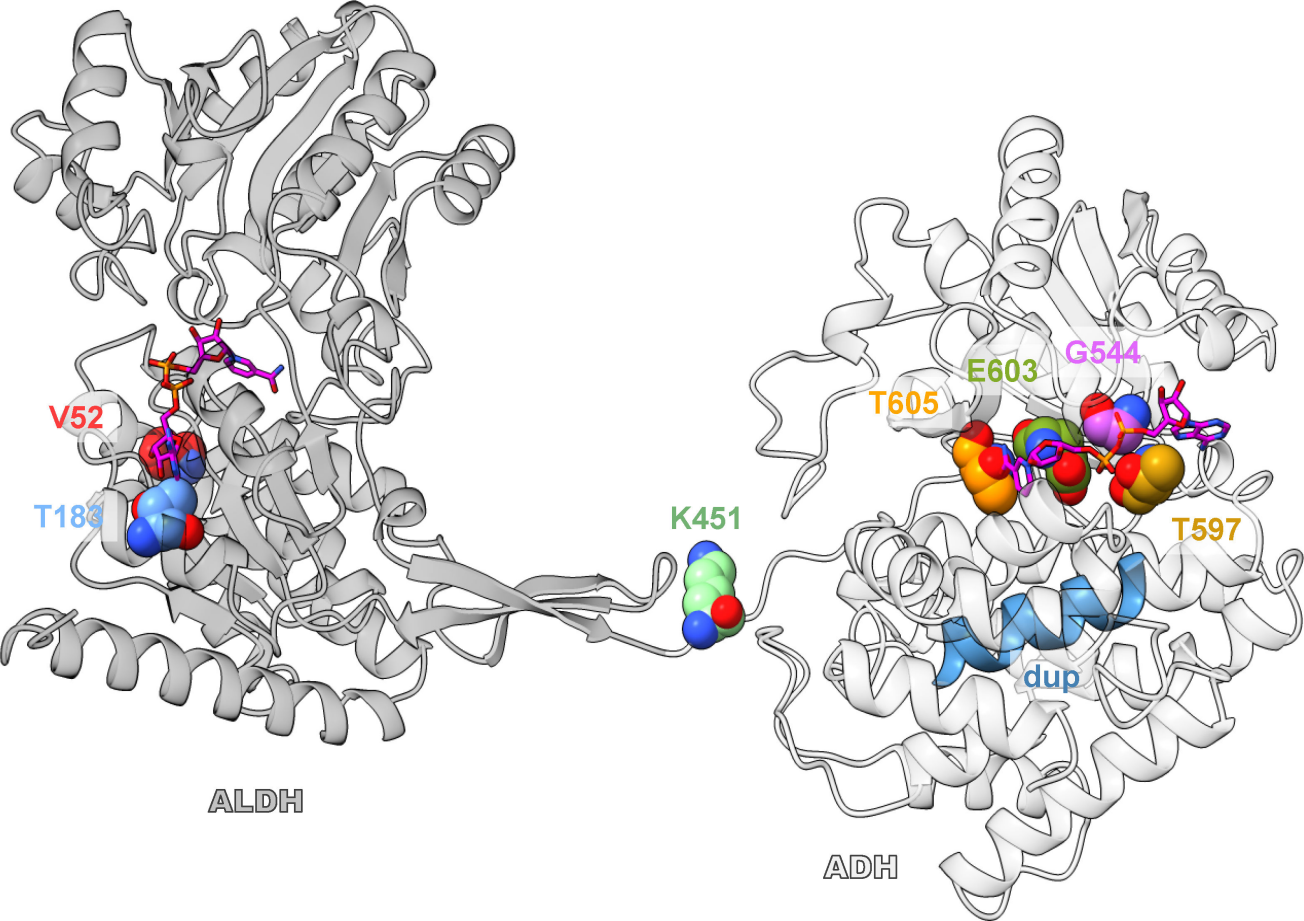
Overview of a monomer of AdhE with mutants shown in spheres, colored based on Fig. 2. The ALDH domain is dark gray, and the ADH domain is white. S602 is not shown due to being obscured by E603.

To understand how mutations in the AdhE enzyme could have affected the activity, we investigated the mutations in the context of a 3D structure of the AdhE protein (Supporting Data set D1). One group of mutations (G544D, T597I, T597K, S602L, and T605I) has a relatively straightforward structural interpretation. All of these mutations line the NADH-binding pocket in the ADH domain of AdhE (Fig. 5) and appear to decrease the ADH activity by interfering with NADH binding.

**Fig 6:**
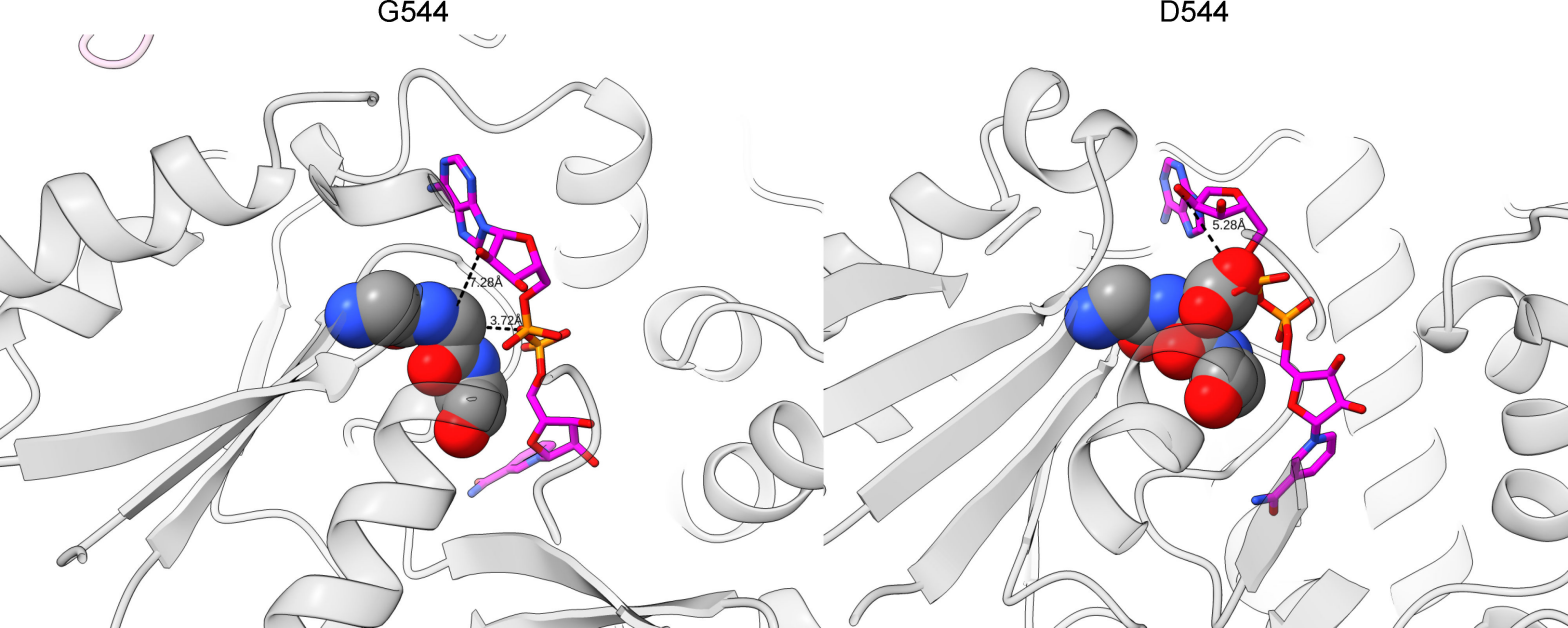
Examining the effect of G544D. The left panel shows the WT AdhE GGG motif (shown as spheres), with the distance measured from the Cɑ of G544 to the pyrophosphate and 2’ OH. The right panel shows the mutant aspartate residue at 544, which causes a clash with the NAD molecule.

The G544 residue is part of a conserved GGG motif, as shown in Fig. 6. The loop of glycines provides a flexible area for the pyrophosphate moiety of the NADH molecule to bind to. When this residue is mutated to aspartate, the pocket is disrupted by a clash at the pyrophosphate moiety. Mutations in this motif in similar proteins in other organisms have been shown to reduce NAD(H) binding in FucO of *E. coli* (53) and reduce the ADH activity in AdhE of *C. thermocellum* (two different mutations, including G552D and G553R) (37).

**Fig 7:**
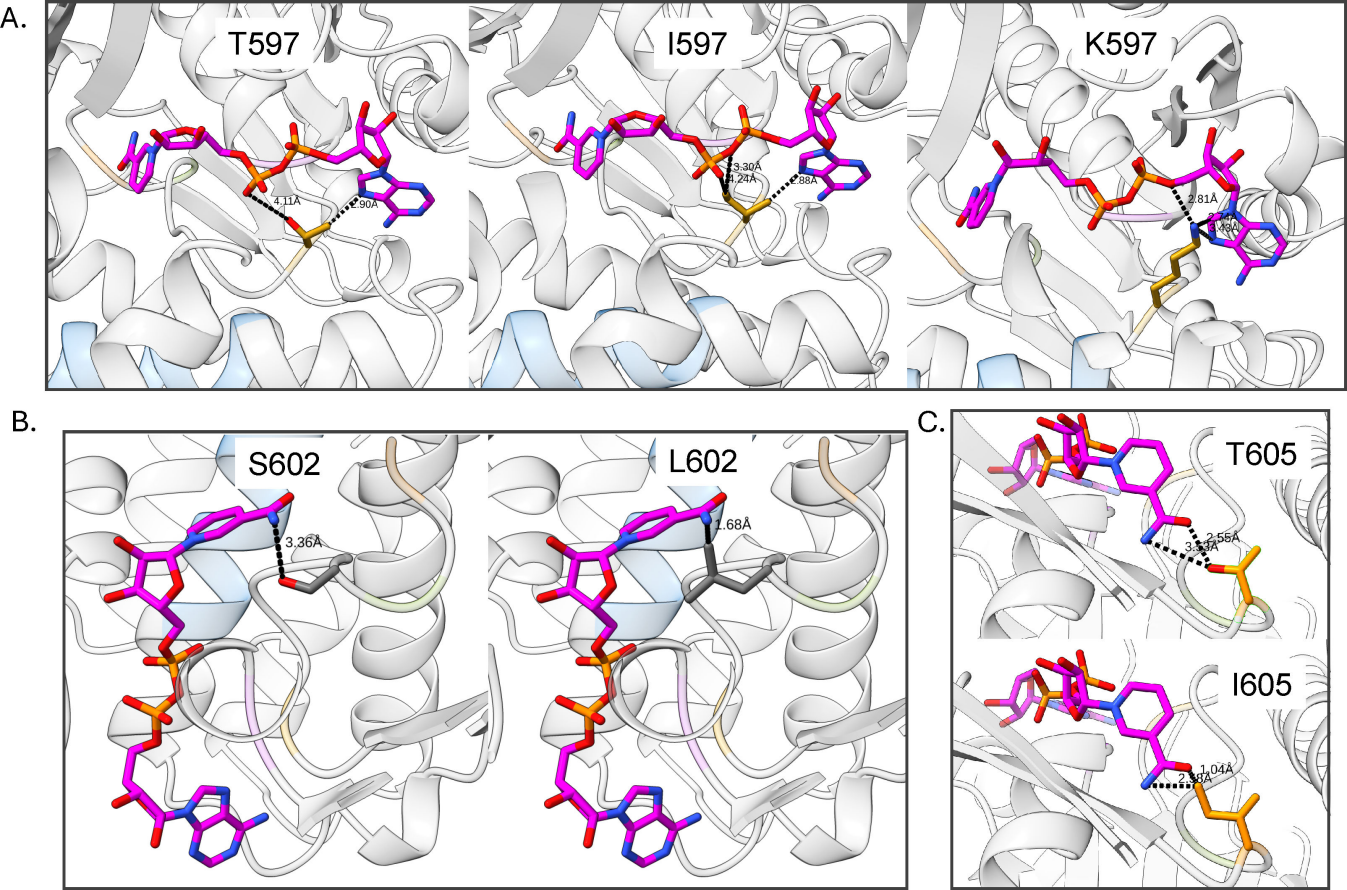
ADH mutants that affect the NAD molecule. (**A**) T597 interacts with the pyrophosphate and adenine moieties of NAD, isoleucine tightens the pocket, and lysine causes a clash at the adenine. (**B**) S602 forms a hydrogen bond with the nicotinamide, while leucine clashes with the nicotinamide. (**C**) T605 forms a hydrogen bond with the nicotinamide, while isoleucine clashes with the same region.

The T597 residue sits between the pyrophosphate and adenine moieties of the NAD^+^ cofactor (Fig. 7A). The isoleucine mutant changes the shape of the pocket, making it much tighter for the NAD^+^, without disrupting the NAD^+^ binding. Conversely, the T597K mutant introduces a much larger side chain, which clashes with the adenine moiety, thereby disrupting the NAD^+^ pocket. At the other end of the NAD^+^ molecule, both the S602 and T605 residues interact with the nicotinamide moiety, with both residues having the capacity to form a hydrogen bond with the NH_2_ group, as shown by the measurements in Fig. 7B and C. Both the leucine (at position 602) and isoleucine (at position 605) mutants disrupt this hydrogen bond and introduce a clash with the nicotinamide moiety.

Due to the complexity of the duplication mutant which occurs on an ɑ-helix in the ADH domain, we modeled it with AlphaFold, which gave an output of five potential structures. Three of the models had a moderately extended helix, leading into a flexible loop, one had a maximally extended helix, and the last model was not structurally feasible. When aligning these structures to the WT AdhE model, the ALDH domain aligns with <1 Å RMSD; however, the ADH domain was approximately 3.5 Å RMSD from the WT ADH domain. This shift was enough to create clashes in the tetramer interface and shift the NADH-binding pocket.

The other mutants in this study had less immediate evidence for why they affected the AdhE activity. The V52 residue is located in the ALDH domain. In the 3D structure, this places it near the part of a flexible loop in the ADH domain of an adjacent monomer (loop 2, as identified by Pony et al. [54]). However, interactions with this loop seem unlikely in this conformation since the valine residue is over 5 Å away from any amino acid in the ADH domain, while the alanine mutant is even more distant. The lack of evidence for changes to structure or binding domains in AdhE is consistent with our enzyme assay data showing no changes in the enzymatic activity (Fig. 2), and we therefore suspect that this mutation is neutral with respect to both structure and function.

K451 is in the linker region between the ALDH and ADH domains, which is associated with the dimerization interface (which is important for spirosome formation). Although it does not appear to play a key role in dimerization (the only interaction it has with a neighboring AdhE protein is through the backbone nitrogen), it may destabilize spirosome formation, and this could account for the slight decrease in the ALDH activity (Fig. 2).

We note that none of the AdhE mutations in the ADH domain are in close proximity to the 2’ OH of the ribose. Since this region is what distinguishes NADH from NADPH, it suggests that none of these mutations would affect the cofactor specificity of the ADH domain of AdhE for NADH vs NADPH, and this is consistent with our enzymatic activity measurements (Fig. 2).

### Dynamic effects of AdhE mutations

**Fig 8:**
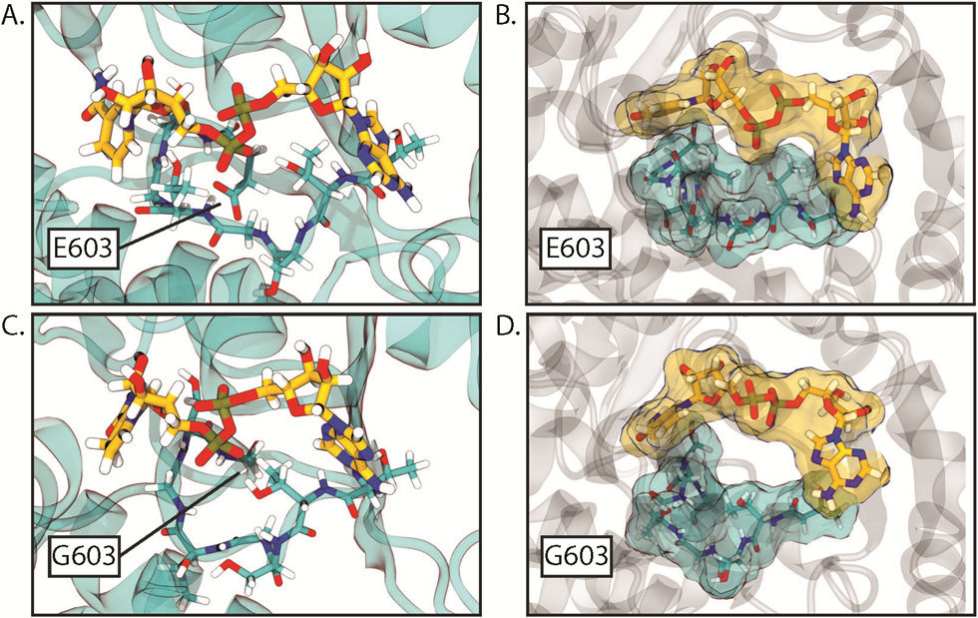
Comparison of effects on residues 597 to 603 (in teal) of the E603G mutation from molecular dynamics simulations, with NADH bound (in yellow). Panels (**A**) and (**B**) depict ball-and-stick and space-filling representations, respectively, of the native E603 structure. Panels (**C**) and (**D**) depict the changes resulting from mutation to G603 after 60 ps and 40 ps of simulation, respectively. The overall structural change in the protein remained stable over the course of the simulations thereafter.

To further study the effect of AdhE mutations, we performed dynamic structural analysis of the ADH domain using MD simulations based on the predicted structure from AlphaFold 2. The purpose of this analysis was to identify second-order effects of mutations that are not well explained by static structural analysis, for example, with the E603G mutation, which exhibited a clear decrease in the ADH activity (Fig. 2), but did not seem to interact directly with NADH within the binding pocket based on the static analysis alone. In this case, dynamic analysis showed that the E603G mutation disrupted the NADH-binding pocket via a second-order interaction with the backbone NH group of T600. The native interaction stabilizes a particular fold of the loop formed by residues 597 to 603 that helps form part of the NADH-binding pocket. The E603G mutation results in the loop swinging open, disrupting several interactions that directly stabilize NADH, as shown in Fig. 8.

For the other point mutations that clearly disrupt the NADH-binding pocket in the structural analysis of the ADH domain (G544D, T597I, T597K, S602L, and T605I), the dynamic analysis results corroborated the static analysis results. Compared to the wild-type, where the NADH cofactor inserted into the binding pocket remained highly stable, each of the disrupting mutations quickly resulted in either a significant perturbation of the binding conformation of NADH or its ejection from the binding site entirely. Representative animations of MD trajectories depicting the dynamic effects of each mutation are available as supporting information (Supporting Datasets D2).

## DISCUSSION

*T. saccharolyticum* is an excellent example of an organism that has been engineered for ethanol production at high titer and yield (19, 20, 24), and it is therefore instructive to try to better understand the mechanisms that have allowed this phenotype. The first steps of engineering *T. saccharolyticum* for increased ethanol production involved disruption of the formation of the byproducts lactate and acetate (19, 24). Subsequent serial transfer for improved growth resulted in mutations concentrated in two genetic regions: the *hfsABCD* operon and *adhE* (25). The mutations in the *hfs* operon appear to act via a regulatory mechanism to increase ethanol production. In fact, deletion of the *hfsA* or *hfsB* genes is sufficient to generate a high-ethanol yield phenotype, even in the presence of functional pathways for lactate and acetate production (55). The purpose of the *adhE* mutations has been harder to explain since reintroduction of these mutations into the WT strain does not significantly affect ethanol production (Fig. 3) (25). We propose that the main function of these mutations is to increase ethanol tolerance by eliminating NADH-linked ADH activity. When the ADH reaction is NADH-linked, a thermodynamic linkage is created between the ADH and glyceraldehyde-3-phosphate dehydrogenase (GAPDH) reactions (due to sharing the same NADH/NAD + cofactor pool) (56). With increase in ethanol titers, the cell can reach a state where it is impossible to have a forward flux through both the ADH and GAPDH reactions simultaneously, blocking fermentation and growth. This hypothesis is presented in detail in other publications from our group (37, 57, 58).

For many years, a prevailing hypothesis in the field of microbial alcohol tolerance is that it is a complex phenotype that is affected by many different genes, with only small contributions from any individual mutation (59–62). Here, we have identified a significant counterexample: in *T. saccharolyticum*, a variety of individual point mutations in the *adhE* gene are each capable of roughly doubling ethanol tolerance in this organism, from 20 g/L to 40 g/L (Fig. 4). This mechanism of ethanol tolerance adaptation has been extensively documented for *C. thermocellum* (37, 38, 51, 52, 63), and there is some evidence for this mechanism in *Thermoanaerobacter thermohydrosulfuricus* (64).

There has been much speculation about the cofactor specificity of the ALDH and ADH reactions mediated by the *T. saccharolyticum* AdhE enzyme. It has previously been proposed that the ALDH activity in *T. saccharolyticum* was either predominantly NADPH-linked (65) or partially NADPH-linked (26). However, in both cases, the ALDH activity was very low. Following the example of the Thauer group (66), we recently showed that adding magnesium to the ALDH enzyme assay buffer dramatically increased the ALDH activity (27). In the presence of 5 mM magnesium, the ALDH activity (both for WT *T. saccharolyticum* and all of the mutants tested) is strictly NADH-linked in both the cell-free extract (Fig. 1) and in purified AdhE proteins (Fig. 2). It is not known why magnesium affects ALDH activity. Interestingly, the activating effect of magnesium appears to be specific to AdhE from *T. saccharolyticum*. Magnesium does not affect the ALDH activity in *C. thermocellum*, and its effect has not been tested in AdhE enzymes from other organisms.

Additional evidence about the role of AdhE mutations can be gathered by looking at the impact of these mutations on the 3D structure of the AdhE protein. There are several examples of mutations that affect the cofactor specificity of ADH enzymes from a variety of organisms. A D494G mutation in *C. thermocellum* AdhE was shown to increase the NADPH-linked ADH activity (26, 37). This mutation corresponds to a D38N mutation on the AdhB enzyme from *Zymomonas mobilis* (67), a D39G mutation from the FucO enzyme in *E. coli* (53), and also corresponds to one of three positions recommended by the heuristic CSR-SALAD algorithm, whose purpose is to identify mutations that change oxidoreductase cofactor specificity (68). What all of these have in common is that the targeted residue is in close proximity to the 2’ hydroxyl moiety of the NAD(H) cofactor, and the mutation provides extra room for the larger 2’ phosphate moiety found in the NADP(H) cofactor. This position corresponds to residue D486 in *T. saccharolyticum*, suggesting that the AdhE enzyme in the WT strain has NADH-linked activity. Although we have previously suggested that the G544D mutation in *T. saccharolyticum* AdhE could change the cofactor specificity via second-order interactions with the D486 residue (26), we now think this is unlikely based on our improved understanding of the AdhE structure (Fig. 6).

Another line of evidence supporting the effect of ADH mutations comes from their interchangeable effect on ethanol production and tolerance. It is very easy for a mutation to disrupt the function of a protein (e.g., disrupt ADH activity) than to acquire a new function (e.g., allow new NADPH-linked ADH activity). Therefore, our observation that the G544D mutation is approximately equivalent to T597K, T597I, and T605I mutations with respect to ethanol production (Fig. 2) and ethanol tolerance (Fig. 3) provides additional evidence that these mutations primarily function by reducing the ADH activity. Together, these three lines of evidence (enzyme assays, structural evidence, and comparison of fermentation phenotypes) suggest that the primary effect of the AdhE G544D mutation in the homoethanologenic strain of *T. saccharolyticum* is to reduce NADH-linked ADH activity.

We will now consider the implications of this modification on the stoichiometry of the pyruvate-to-ethanol conversion pathway. In the homoethanologen strain of *T. saccharolyticum*, the ALDH reaction is mediated by the AdhE enzyme and is NADH-linked. The ADH reaction is not mediated by AdhE (since the G544D mutation eliminates much of the activity of the ADH domain) and is instead mediated by the AdhA enzyme (35). Thus, each molecule of acetyl-CoA that is converted to ethanol requires one NADH and one NADPH. The glycolysis and fermentation pathways of *T. saccharolyticum* produce one NADH and one reduced ferredoxin for each half-glucose that is converted to acetyl-CoA. A stoichiometrically balanced ethanol production pathway requires a reaction or set of reactions that can transfer electrons from reduced ferredoxin to NADP^+^; however, the enzyme(s) that mediate this reaction are not known. It has recently been proposed that simultaneous production and consumption of hydrogen (i.e., hydrogen cycling) may account for this phenomenon (69), but additional research is likely necessary to confirm.

The mutations in the ADH domain of AdhE also have implications for our understanding of substrate channeling. Substrate channeling involves the direct transfer of the products of one reaction to the active site of a second reaction. The bifunctional structure of AdhE has inspired speculation that substrate channeling may be present in AdhE (54, 70–73). However, in the homoethanologenic strain of *T. saccharolyticum* (M1442), the acetaldehyde produced by the ALDH domain of AdhE is converted to ethanol by the monofunctional AdhA enzyme (Fig. 3, Supporting Information Fig. S1), implying that substrate channeling is not necessary for the high-titer ethanol production (~70 g/L) (20) demonstrated by this strain. The presence of substrate channeling in WT AdhE, however, remains an open question.

## SUPPLEMENTAL MATERIAL

Dataset D1 jb.00015-25-s0001.txt PDB model of *T. saccharolyticum* AdhE.

Supplemental tables and figure jb.00015-25-s0002.docx Tables S1 to S4 and Figure S1.

Dataset D2 - G544D jb.00015-25-s0003.mp4 Animations of molecular dynamics trajectories for the AdhE G544D mutant.

Dataset D2 - S602L jb.00015-25-s0004.mp4 Animations of molecular dynamics trajectories for the AdhE S602L mutant. Animations of molecular dynamics trajectories for the AdhE G544D mutant.

Dataset D2 - T597I jb.00015-25-s0005.mp4 Animations of molecular dynamics trajectories for the AdhE T597I mutant.

Dataset D2 - T597K jb.00015-25-s0006.mp4 Animations of molecular dynamics trajectories for the AdhE T597K mutant.

Dataset D2 - T605I jb.00015-25-s0007.mp4 Animations of molecular dynamics trajectories for the AdhE T605I mutant.

ASM does not own the copyrights to Supplemental Material that may be linked to, or accessed through, an article. The authors have granted ASM a non-exclusive, world-wide license to publish the Supplemental Material files. Please contact the corresponding author directly for reuse.
