# Supplementary material for: The role of AdhE mutations in Thermoanaerobacterium saccharolyticum
Source: J Bacteriol. 2025 Apr 30;207(5):e00015-25. doi: 10.1128/jb.00015-25 (PMC12096837; doi:10.1128/jb.00015-25)
Supplement: Supplemental tables and figure — Tables S1 to S4 and Figure S1. [file jb.00015-25-s0002.docx]

# Supporting information

The Role of AdhE Mutations in *Thermoanaerobacterium saccharolyticum*

João Henrique T. M. Fabri ^1^, Angel Pech-Canul ^2,3^, Samantha J. Ziegler ^3,4^, Tucker Emme Burgin ^2^, Isaiah D. Richardson ^2,3^, Marybeth I. Maloney ^2,3^, Yannick J. Bomble ^3,4^, Lee R. Lynd ^1,2,3,5^, Daniel G. Olson ^1,2,3,*^

# Affiliations

^1^ Centro de Biologia Molecular e Engenharia Genética (CBMEG), Universidade Estadual de Campinas (UNICAMP), Campinas,SP, Brazil

^2^ Thayer School of Engineering, Dartmouth College, Hanover, NH, United States

^3^ Center for Bioenergy Innovation, Oak Ridge National Laboratory, Oak Ridge, TN

^4^ Biosciences Center, National Renewable Energy Laboratory, Golden, CO, 80401

^5^ Terragia Corporation, Hanover, NH, United States

^*^ To whom correspondence should be addressed: daniel.g.olson@dartmouth.edu

**Supporting Table S1**. Primers used for *T. saccharolyticum* transformation and validation

| **Primer** | **Sequence** | **Purpose** |
| --- | --- | --- |
| adhE 5’ FW | TACATCGTCCAATCTTTTGT | Amplify adhE 5' homology site |
| adhE 1765 REV | CGGTATTGCGATGAATAGCG | Amplify adhE gene |
| adhE AAA FW | TATTCATCGCAATACCGACAAAAAGCGGTACAGGCTCAGAAGTGACAGCA | Amplify adhE with the T597K mutation |
| adhE ORF REV | TCAAATGGTTCGCTGGGTTTTAAGCCGTTCTTTTTCTTAAG | Amplify adhE gene |
| adhE 3' FW | ATGAAGTTCCTCGTACATAGCAAGTTAATTAAAACTTATAAGCC | Amplify adhE 3' homology site |
| adhE 3’ REV | ACAGACTTTACACGACTGG | Amplify adhE 3' homology site |
| adhE ext FW | GCCTTTGCCTTAAAAATACA | Amplify adhE external genomic fragment |
| adhE ext REV | TCGATGTTATGGCGCTTT | Amplify adhE external genomic fragment |
| adhE seq FW | AATTCGTACAATCCTGACTT | Sequencing adhE locus |
| adhE seq REV | GATGCCATTACTGATACGTA | Sequencing adhE locus |
| adhE 1755 REV | GGTATTGCGATGAATAGCGCTTTTTTGCC | Amplify adhE gene |
| adhE ATA FW | GCGCTATTCATCGCAATACCGACAATAAGCGGTACAGGCTCAGAAGTGACAGCA | Amplify adhE with the T597I mutation |
| adhE 1791 REV | ACTTCTGAGCCTGTGCCGCT | Amplify adhE gene |
| adhE ATA 2 FW | GCGGCACAGGCTCAGAAGTGATAGCTTTTGCCGTAATAACCGACAA | Amplify adhE with the T605I mutation |
| adhE check FW RsaI | ATCCTGACTTAATAATCGCT | Used for validation with restriction enzyme |
| adhE check REV AluI | ACCTTATTACATACGGCAGA | Used for validation with restriction enzyme |
| adhE check FW AluI | GCGAAGATTGCTGAAATG | Used for validation with restriction enzyme |
| adhE check REV AluI | ACCTTATTACATACGGCAGA | Used for validation with restriction enzyme |
| adhE GGA FW | GCGCTATTCATCGCAATACCGACAACAAGCGGTACAGGCTCAGGAGTGACAGCATTTGCCGTAA | Amplify adhE with the E603G mutation |
| adhE 1600 FW | TAAGTCAGGATTGTACGAAT | Amplify adhE gene |
| adhE wt FW | AATTCGTACAATCCTGACTTAATAATAGCTGTAGGCGGTGGCTCTGCAATA | Amplify adhE gene |
| adhE wt 2 FW | AATTCGTACAATCCTGACTTAATAATAGCTGTAGGCGATGGCTCTGCAATAGACGCAGCA | Amplify adhE gene |

**Supporting Table S2**. Batch Fermentation Data Endpoint Measurements

| Sample ID | Strain description | Glucose (mM) | Cellobiose remaining (mM) | Cellobiose consumed (mM) | Pyruvate (mM) | Malate (mM) | Succinate (mM) | Lactate (mM) | Formate (mM) | Acetate (mM) | Ethanol (mM) |
| --- | --- | --- | --- | --- | --- | --- | --- | --- | --- | --- | --- |
| M1442 1 | LL1049 | 0.856 | 0 | 13.812 | 0.539 | 0 | 0.06 | 0 | 7.457 | 1.237 | 49.474 |
| M1442 2 | LL1049 | 0.956 | 0 | 13.812 | 0.555 | 0 | 0 | 0 | 8.243 | 1.100 | 49.345 |
| M1442 3 | LL1049 | 0.916 | 0 | 13.812 | 0.548 | 0 | 0 | 0 | 8.031 | 1.203 | 49.654 |
| LL1287 1 | ∆adhA | 0.706 | 0 | 13.812 | 0.524 | 0.550 | 0 | 0 | 4.74 | 0.264 | 23.826 |
| LL1287 2 | ∆adhA | 1.017 | 0 | 13.812 | 0.593 | 0.591 | 0 | 0 | 4.514 | 0.355 | 23.247 |
| LL1287 3 | ∆adhA | 0.766 | 0 | 13.812 | 0.533 | 0.529 | 0 | 0 | 4.489 | 0.242 | 23.589 |
| LL1025 1 | wild-type | 0.868 | 0 | 13.812 | 0.690 | 0.606 | 0 | 2.550 | 0 | 15.978 | 36.257 |
| LL1025 2 | wild-type | 0.854 | 0 | 13.812 | 0.777 | 0.597 | 0 | 2.669 | 0 | 15.602 | 36.594 |
| LL1025 3 | wild-type | 0.899 | 0 | 13.812 | 0.498 | 0.587 | 0 | 2.835 | 0 | 15.436 | 36.454 |
| A2G0001 1 | adhE^T597K^ | 1.014 | 0 | 13.812 | 0.645 | 0 | 0 | 0 | 6.980 | 0.987 | 49.971 |
| A2G0001 2 | adhE^T597K^ | 0.728 | 0 | 13.812 | 0.583 | 0 | 0 | 0 | 7.181 | 1.216 | 50.491 |
| A2G0001 3 | adhE^T597K^ | 0.605 | 0 | 13.812 | 0.666 | 0 | 0 | 0 | 7.024 | 1.241 | 51.190 |
| A2G0001 4 | adhE^T597K^ | 0.799 | 0 | 13.812 | 0.527 | 0 | 0 | 0 | 6.869 | 1.208 | 51.088 |
| A2G0001 5 | adhE^T597K^ | 0.547 | 0 | 13.812 | 0.694 | 0 | 0 | 0 | 6.974 | 1.080 | 51.101 |
| A2G0001 6 | adhE^T597K^ | 0.711 | 0 | 13.812 | 0.600 | 0 | 0 | 0 | 6.638 | 1.231 | 51.136 |
| A2G0002 1 | adhE^T597I^ | 0.862 | 0 | 13.812 | 0.599 | 0 | 0.074 | 0 | 5.461 | 0.941 | 51.310 |
| A2G0002 2 | adhE^T597I^ | 0.682 | 0 | 13.812 | 0.593 | 0 | 0 | 0 | 5.019 | 0.984 | 51.658 |
| A2G0002 3 | adhE^T597I^ | 0.738 | 0 | 13.812 | 0.574 | 0 | 0 | 0 | 6.051 | 1.149 | 51.621 |
| A2G0002 4 | adhE^T597I^ | 0.747 | 0 | 13.812 | 0.579 | 0 | 0.088 | 0 | 6.124 | 1.008 | 51.741 |
| A2G0002 5 | adhE^T597I^ | 0.717 | 0 | 13.812 | 0.580 | 0 | 0.04 | 0 | 6.156 | 1.257 | 51.651 |
| A2G0003 1 | adhE^T605I^ | 0.6 | 0 | 13.812 | 0.869 | 0 | 0 | 0 | 5.911 | 0.952 | 49.323 |
| A2G0003 2 | adhE^T605I^ | 0.597 | 0 | 13.812 | 0.876 | 0 | 0 | 0 | 6.655 | 1.211 | 48.399 |
| A2G0003 3 | adhE^T605I^ | 0.75 | 0 | 13.812 | 0.725 | 0 | 0 | 0 | 6.938 | 0.950 | 49.915 |
| A2G0003 4 | adhE^T605I^ | 0.721 | 0 | 13.812 | 0.700 | 0 | 0 | 0 | 6.894 | 1.020 | 49.332 |
| A2G0004 1 | adhE^E603G^ | 0.733 | 0 | 13.812 | 0.492 | 0 | 0 | 0 | 6.946 | 1.184 | 52.106 |
| A2G0004 2 | adhE^E603G^ | 0.709 | 0 | 13.812 | 0.505 | 0 | 0.066 | 0 | 5.916 | 1.047 | 51.336 |
| A2G0004 3 | adhE^E603G^ | 0.685 | 0 | 13.812 | 0.502 | 0 | 0.016 | 0.038 | 6.032 | 1.043 | 51.858 |
| A2G0004 4 | adhE^E603G^ | 0.708 | 0 | 13.812 | 0.509 | 0 | 0 | 0 | 6.162 | 1.007 | 51.730 |
| A2G0004 5 | adhE^E603G^ | 0.726 | 0 | 13.812 | 0.534 | 0 | 0 | 0 | 6.399 | 1.210 | 52.344 |
| A2G0022 1 | wild-type adhE^WT^ | 0.661 | 0 | 13.812 | 0.549 | 0 | 0 | 1.033 | 0 | 18.115 | 37.380 |
| A2G0022 2 | wild-type adhE^WT^ | 0.653 | 0 | 13.812 | 0.694 | 0 | 0 | 1.335 | 0 | 18.224 | 37.133 |
| A2G0022 3 | wild-type adhE^WT^ | 1.108 | 0 | 13.812 | 0.430 | 0 | 0 | 1.295 | 0 | 15.008 | 37.133 |
| A2G0023 1 | wild-type adhE^G544D^ | 0.602 | 0 | 13.812 | 0.459 | 0 | 0 | 1.659 | 0 | 14.304 | 40.219 |
| A2G0023 2 | wild-type adhE^G544D^ | 0.725 | 0 | 13.812 | 0.689 | 0 | 0 | 1.202 | 0 | 13.665 | 39.345 |
| A2G0023 3 | wild-type adhE^G544D^ | 0.728 | 0 | 13.812 | 0.720 | 0 | 0 | 1.471 | 0 | 13.746 | 39.719 |
| A2G0023 4 | wild-type adhE^G544D^ | 0.673 | 0 | 13.812 | 0.839 | 0 | 0 | 2.166 | 0 | 13.538 | 38.787 |
| A2G0023 5 | wild-type adhE^G544D^ | 0.611 | 0 | 13.812 | 0.779 | 0 | 0 | 3.874 | 0 | 12.916 | 36.695 |
| A2G0024 1 | LL1049 adhE^WT^ | 1.36 | 0 | 13.812 | 1.706 | 0 | 0 | 0 | 7.13 | 0.939 | 46.687 |
| A2G0024 2 | LL1049 adhE^WT^ | 1.264 | 0 | 13.812 | 1.756 | 0 | 0 | 0 | 6.743 | 1.121 | 47.428 |
| A2G0024 3 | LL1049 adhE^WT^ | 0.872 | 0 | 13.812 | 0.881 | 1.346 | 0 | 0 | 8.813 | 0.788 | 50.022 |
| A2G0024 4 | LL1049 adhE^WT^ | 0.71 | 0 | 13.812 | 0.798 | 1.226 | 0 | 0 | 8.772 | 0.916 | 49.584 |
| A2G0025 1 | LL1049 adhE^G544D^ | 0.962 | 0 | 13.812 | 0.794 | 0 | 0 | 0 | 9.463 | 0.77 | 51.307 |
| A2G0025 2 | LL1049 adhE^G544D^ | 0.939 | 0 | 13.812 | 0.832 | 0 | 0 | 0 | 8.920 | 0.833 | 52.040 |
| A2G0025 3 | LL1049 adhE^G544D^ | 0.91 | 0 | 13.812 | 0.749 | 0 | 0 | 0 | 9.417 | 0.719 | 52.284 |
| A2G0025 4 | LL1049 adhE^G544D^ | 0.923 | 0 | 13.812 | 0.811 | 0 | 0 | 0 | 9.743 | 0.853 | 52.293 |
| A2G0025 5 | LL1049 adhE^G544D^ | 0.982 | 0 | 13.812 | 0.839 | 0 | 0 | 0 | 9.583 | 0.911 | 51.327 |
| Medium |  | 0.108 | 13.812 |  | 0 | 0 | 0 | 0 | 0 | 0 | 0 |

**Supporting Table S3**. Ethanol tolerance assay. Growth rates (uMax) are presented for various strains at various ethanol concentrations.

| **Strain** | **Ethanol concentration (g/L)** | | | | | | | |
| --- | --- | --- | --- | --- | --- | --- | --- | --- |
|  | **0** | **5** | **10** | **20** | **30** | **40** | **50** | **60** |
| **M1442 1** | 0,445 | 0,428 | 0,41 | 0,35 | 0,124 | 0,142 | 0 | 0 |
| **M1442 2** | 0,356 | 0,444 | 0,342 | 0,231 | 0,097 | 0,039 | 0 | 0 |
| **M1442 3** | 0,346 | 0,427 | 0,346 | 0,151 | 0,09 | 0,033 | 0 | 0 |
| **M1442 4** | 0,481 | 0,44 | 0,414 | 0,268 | 0,138 | 0,002 | 0 | 0 |
| **M1442 5** | 0,385 | 0,468 | 0,411 | 0,303 | 0,243 | 0 | 0 | 0 |
| **M1442 6** | 0,318 | 0,366 | 0,308 | 0,201 | 0,198 | 0,148 | 0 | 0 |
| **M1442 7** | 0,34 | 0,271 | 0,284 | 0,146 | 0,135 | 0 | 0 | 0 |
| **M1442 8** | 0,501 | 0,424 | 0,236 | 0,186 | 0,119 | 0 | 0 | 0 |
| **M1442 9** | 0,471 | 0,489 | 0,438 | 0,33 | 0,194 | 0 | 0 | 0 |
| **M1442 10** | 0,31 | 0,348 | 0,337 | 0,266 | 0,143 | 0,056 | 0 | 0 |
| **LL1025 1** | 0,184 | 0,174 | 0,181 | 0,077 | 0 | 0 | 0 | 0 |
| **LL1025 2** | 0,158 | 0,209 | 0,158 | 0,1 | 0 | 0 | 0 | 0 |
| **LL1025 3** | 0,188 | 0,077 | 0,061 | 0,035 | 0,027 | 0 | 0 | 0 |
| **LL1025 4** | 0,188 | 0,162 | 0,116 | 0,09 | 0,018 | 0 | 0 | 0 |
| **LL1025 5** | 0,175 | 0,143 | 0,104 | 0,05 | 0,028 | 0 | 0 | 0 |
| **LL1025 6** | 0,236 | 0,151 | 0,137 | 0 | 0 | 0 | 0 | 0 |
| **LL1025 7** | 0,178 | 0,183 | 0,113 | 0 | 0 | 0 | 0 | 0 |
| **LL1025 8** | 0,276 | 0,226 | 0,155 | 0 | 0 | 0 | 0 | 0 |
| **LL1025 9** | 0,27 | 0,244 | 0,262 | 0 | 0 | 0 | 0 | 0 |
| **A2G0001 1** | 0,407 | 0,395 | 0,307 | 0,131 | 0,102 | 0,056 | 0 | 0 |
| **A2G0001 2** | 0,371 | 0,443 | 0,441 | 0,25 | 0,181 | 0,044 | 0 | 0 |
| **A2G0001 3** | 0,263 | 0,266 | 0,193 | 0,11 | 0,043 | 0,046 | 0 | 0 |
| **A2G0001 4** | 0,353 | 0,389 | 0,335 | 0,242 | 0,174 | 0 | 0 | 0 |
| **A2G0001 5** | 0,384 | 0,425 | 0,359 | 0,272 | 0,16 | 0,115 | 0 | 0 |
| **A2G0001 6** | 0,374 | 0,372 | 0,364 | 0,18 | 0,127 | 0,032 | 0 | 0 |
| **A2G0002 1** | 0,411 | 0,421 | 0,39 | 0,084 | 0,052 | 0 | 0 | 0 |
| **A2G0002 2** | 0,366 | 0,398 | 0,271 | 0,23 | 0,078 | 0 | 0 | 0 |
| **A2G0002 3** | 0,351 | 0,41 | 0,312 | 0,344 | 0,159 | 0,029 | 0 | 0 |
| **A2G0002 4** | 0,358 | 0,255 | 0,35 | 0,076 | 0,053 | 0 | 0 | 0 |
| **A2G0002 5** | 0,376 | 0,402 | 0,277 | 0,185 | 0,146 | 0 | 0 | 0 |
| **A2G0002 6** | 0,377 | 0,384 | 0,326 | 0,113 | 0,113 | 0 | 0 | 0 |
| **A2G0002 7** | 0,372 | 0,416 | 0,404 | 0,167 | 0,105 | 0 | 0 | 0 |
| **A2G0002 8** | 0,369 | 0,414 | 0,411 | 0,173 | 0,082 | 0 | 0 | 0 |
| **A2G0003 1** | 0,371 | 0,417 | 0,307 | 0,094 | 0,067 | 0 | 0 | 0 |
| **A2G0003 2** | 0,35 | 0,426 | 0,373 | 0,222 | 0,183 | 0 | 0 | 0 |
| **A2G0003 3** | 0,359 | 0,478 | 0,288 | 0,305 | 0,259 | 0 | 0 | 0 |
| **A2G0003 4** | 0,36 | 0,412 | 0,254 | 0,166 | 0,139 | 0 | 0 | 0 |
| **A2G0003 5** | 0,366 | 0,424 | 0,418 | 0,208 | 0,222 | 0,126 | 0 | 0 |
| **A2G0004 1** | 0,238 | 0,207 | 0,193 | 0,192 | 0,154 | 0,023 | 0 | 0 |
| **A2G0004 2** | 0,363 | 0,361 | 0,331 | 0,097 | 0,05 | 0,014 | 0 | 0 |
| **A2G0004 3** | 0,248 | 0,238 | 0,09 | 0,064 | 0,011 | 0 | 0 | 0 |
| **A2G0004 4** | 0,253 | 0,36 | 0,353 | 0,312 | 0,135 | 0 | 0 | 0 |
| **A2G0004 5** | 0,366 | 0,477 | 0,399 | 0,257 | 0,105 | 0,019 | 0 | 0 |
| **A2G0004 6** | 0,211 | 0,166 | 0,151 | 0,121 | 0 | 0 | 0 | 0 |
| **A2G0022 1** | 0,3 | 0,118 | 0,11 | 0 | 0 | 0 | 0 | 0 |
| **A2G0022 2** | 0,335 | 0,113 | 0,08 | 0 | 0 | 0 | 0 | 0 |
| **A2G0022 3** | 0,334 | 0,012 | 0,004 | 0 | 0 | 0 | 0 | 0 |
| **A2G0022 4** | 0,28 | 0,263 | 0,118 | 0 | 0 | 0 | 0 | 0 |
| **A2G0022 5** | 0,33 | 0,176 | 0,059 | 0 | 0 | 0 | 0 | 0 |
| **A2G0022 6** | 0,141 | 0,122 | 0,03 | 0 | 0 | 0 | 0 | 0 |
| **A2G0022 7** | 0,247 | 0,19 | 0,081 | 0 | 0 | 0 | 0 | 0 |
| **A2G0022 8** | 0,264 | 0,209 | 0,109 | 0 | 0 | 0 | 0 | 0 |
| **A2G0023 1** | 0,267 | 0,353 | 0,252 | 0,118 | 0,126 | 0,054 | 0 | 0 |
| **A2G0023 2** | 0,196 | 0,339 | 0,308 | 0,075 | 0,071 | 0,06 | 0 | 0 |
| **A2G0023 3** | 0,198 | 0,366 | 0,309 | 0,134 | 0,08 | 0,059 | 0 | 0 |
| **A2G0023 4** | 0,157 | 0,34 | 0,157 | 0,073 | 0,056 | 0,089 | 0 | 0 |
| **A2G0023 5** | 0,202 | 0,305 | 0,134 | 0,076 | 0,092 | 0,094 | 0 | 0 |
| **A2G0023 6** | 0,199 | 0,374 | 0,11 | 0,08 | 0,071 | 0,091 | 0 | 0 |
| **A2G0023 7** | 0,274 | 0,358 | 0,344 | 0,067 | 0,06 | 0,082 | 0 | 0 |
| **A2G0023 8** | 0,299 | 0,379 | 0,347 | 0,067 | 0,07 | 0,085 | 0 | 0 |
| **A2G0023 9** | 0,285 | 0,399 | 0,292 | 0,062 | 0,078 | 0,07 | 0 | 0 |
| **A2G0023 10** | 0,295 | 0,408 | 0,321 | 0,066 | 0,071 | 0,074 | 0 | 0 |
| **A2G0023 11** | 0,249 | 0,389 | 0,235 | 0,064 | 0,067 | 0,072 | 0 | 0 |
| **A2G0023 12** | 0,356 | 0,343 | 0,256 | 0,072 | 0,077 | 0,129 | 0 | 0 |
| **A2G0023 13** | 0,329 | 0,273 | 0,304 | 0,077 | 0,063 | 0,098 | 0 | 0 |
| **A2G0023 14** | 0,23 | 0,224 | 0,264 | 0,09 | 0,067 | 0,064 | 0 | 0 |
| **A2G0023 15** | 0,299 | 0,387 | 0,259 | 0,157 | 0,093 | 0,044 | 0 | 0 |
| **A2G0023 16** | 0,292 | 0,387 | 0,218 | 0,108 | 0,191 | 0,166 | 0 | 0 |
| **A2G0023 17** | 0,207 | 0,256 | 0,121 | 0,149 | 0,091 | 0,231 | 0 | 0 |
| **A2G0023 18** | 0,178 | 0,37 | 0,1 | 0,156 | 0,14 | 0,06 | 0 | 0 |
| **A2G0024 1** | 0,174 | 0,192 | 0 | 0 | 0 | 0 | 0 | 0 |
| **A2G0024 2** | 0,19 | 0,1 | 0 | 0 | 0 | 0 | 0 | 0 |
| **A2G0024 3** | 0,194 | 0,179 | 0,04 | 0 | 0 | 0 | 0 | 0 |
| **A2G0024 4** | 0,309 | 0,074 | 0 | 0 | 0 | 0 | 0 | 0 |
| **A2G0024 5** | 0,282 | 0 | 0 | 0 | 0 | 0 | 0 | 0 |
| **A2G0024 6** | 0,305 | 0,07 | 0,03 | 0 | 0 | 0 | 0 | 0 |
| **A2G0025 1** | 0,347 | 0,414 | 0,249 | 0,143 | 0,158 | 0 | 0 | 0 |
| **A2G0025 2** | 0,364 | 0,436 | 0,322 | 0,164 | 0 | 0 | 0 | 0 |
| **A2G0025 3** | 0,28 | 0,287 | 0,268 | 0,275 | 0,185 | 0 | 0 | 0 |
| **A2G0025 4** | 0,523 | 0,38 | 0,307 | 0,223 | 0,137 | 0 | 0 | 0 |
| **A2G0025 5** | 0,289 | 0,282 | 0,191 | 0,183 | 0,175 | 0 | 0 | 0 |
| **A2G0025 6** | 0,362 | 0,291 | 0,207 | 0,178 | 0,091 | 0 | 0 | 0 |

**Supporting Table S4**. Effectiveness of insertion of *adhE* mutations

| **Strain** | **Background strain** | **Allele inserted** | **Ratio of correct insertion** | **Reversed mutation (percentage)** |
| --- | --- | --- | --- | --- |
| A2G0022 | LL1025 (WT) | adhE^WT^ + kan | 10/10 (100%) | - |
| A2G0023 | LL1025 (WT) | adhE^G544D^ + kan | 16/20 (80%) | WT (20%) |
| A2G0024 | M1442 (LL1049) | adhE^WT^ + kan | 4/20 (20%) | G544D (80%) |
| A2G0025 | M1442 (LL1049) | adhE^G544D^ + kan | 10/10 (100%) | - |

**Supporting Dataset D1**. PDB model of *T. saccharolyticum* AdhE

**Supporting Dataset D2.** Representative animations of Molecular Dynamics trajectories

**
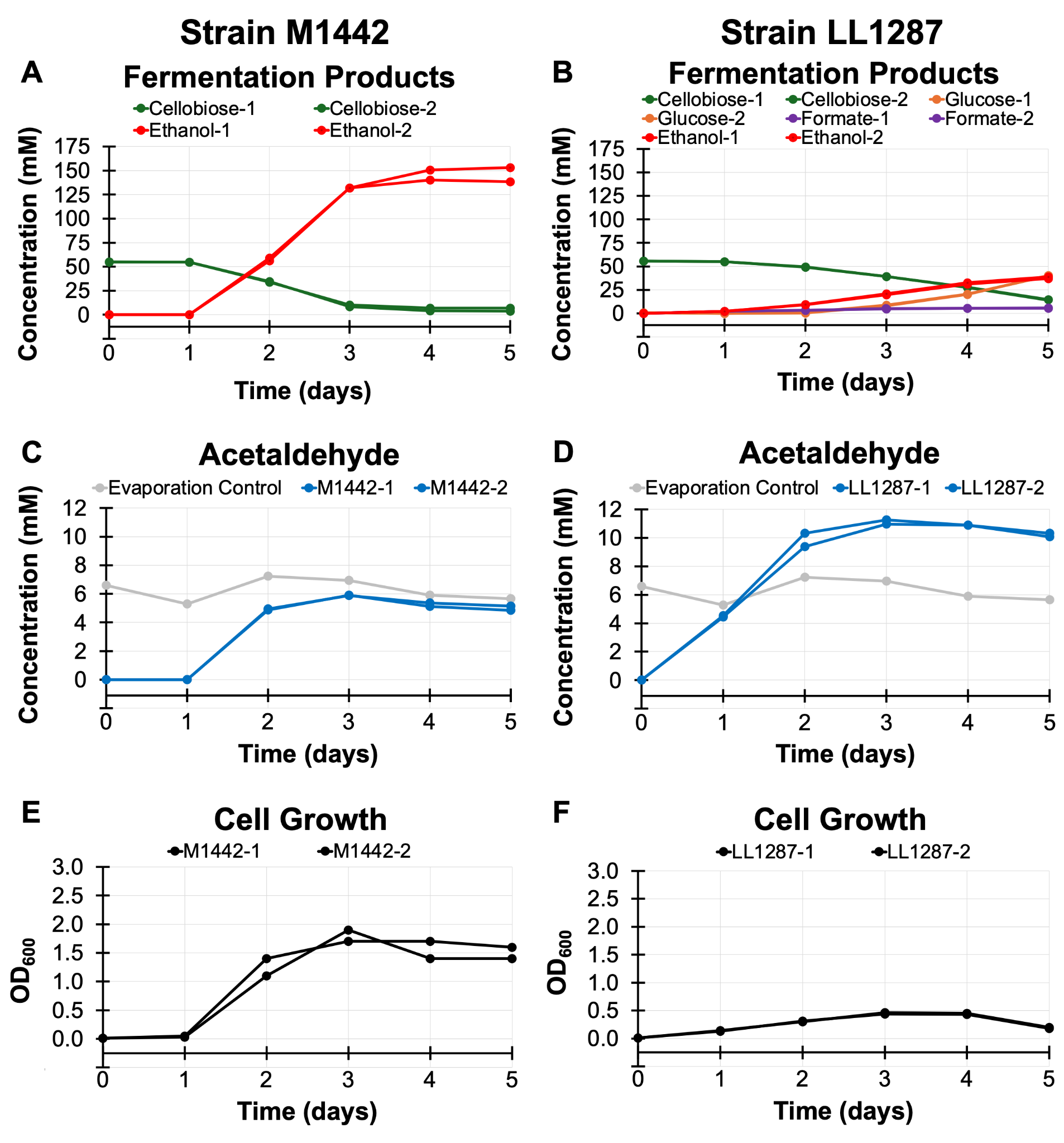
**

**Supporting Figure S1**. Batch fermentations of the homoethanologen strain (M1442) and a descendant of that strain with an additional disruption of the *adhA* gene (LL1287). Fermentations were performed in biological duplicate at 55°C in 30 ml of MTC-6 media with 20 g/L cellobiose (~55 mM) in 50 ml plastic screw-capped tubes (Falcon, Corning Science, Part number 352098) for 5 days. Samples were taken each day to quantify fermentation products (panels A-D) by HPLC system. For acetaldehyde measurements, an uninoculated tube with 10 mM acetaldehyde was prepared as an evaporation control. Acetaldehyde concentration was measured in the supernatant. Cell growth was quantified based on changes in absorbance measured at 600 nm (panels E-F). Data from each replicate is plotted separately. In some cases, the data points overlap due to similarity between replicates.
